# A Scoping Review of How Income Affects Accessing Local Green Space to Engage in Outdoor Physical Activity to Improve Well-Being: Implications for Post-COVID-19

**DOI:** 10.3390/ijerph17249313

**Published:** 2020-12-12

**Authors:** Llinos Haf Spencer, Mary Lynch, Catherine L. Lawrence, Rhiannon Tudor Edwards

**Affiliations:** 1 Centre for Health Economics and Medicine Evaluation, Bangor University, Bangor LL57 2PZ, UK; m.lynch@bangor.ac.uk (M.L.); c.l.lawrence@bangor.ac.uk (C.L.L.); r.t.edwards@bangor.ac.uk (R.T.E.); 2 School of Health Sciences, Bangor University, Bangor LL57 2EF, UK

**Keywords:** valuing nature, public health, physical activity, green spaces, blue spaces, income, socioeconomic status

## Abstract

Background: The National Institute for Health and Care Excellence (NICE) has set out guidance for promoting physical activity (PA) in the physical environment to promote health and well-being. The aim of this selective scoping review was to investigate the influence of gross income on accessing local green spaces to engage in PA and the associated health benefits. Methods: A scoping review was conducted of international literature to facilitate the clarification of the research question. Findings: 15 papers were critically appraised under two themes: (1) environments and well-being and (2) PA and income/socioeconomic status and impact on the frequency, duration and opportunity to engage in PA. Interpretation: Income is related to differential use of green and blue spaces for PA, due mainly to access issues. People who live in lower socioeconomic areas tend to be more sedentary and there are also gender differences related to PA in built environments. Conclusion: There is an effect of income in using green spaces for PA, but the relationship is non-linear, and there is still a lack of knowledge about what kind of green spaces are best for health benefits. The COVID-19 pandemic highlighted the importance of accessing green local spaces to engage in physical exercise to improve well-being among the public.

## 1. Introduction

The World Health Organization (WHO) has a framework for promoting health through physical activity (PA) [1] and the UK National Institute for Health and Care Excellence (NICE) has set out guidance for promoting PA in the physical environment to promote health and well-being [2]. During the COVID-19 pandemic lockdown, the UK Government encouraged households to exercise each day near to their home, making use of urban and rural green space. Leading the way in the UK, the Welsh government has made promoting well-being one of its main aims as set out in the Future Generations Act (2015) [3], and having a healthy and active population is one of the main themes in the “Prosperity for All” Welsh Government national strategy [4].

Technological advances have shifted employment to become more sedentary, and physical movement and contact with the outdoors has been minimised with plausible detrimental effects on the health of workers [5]. However, even jobs which are highly physically demanding, such as nursing and other care work, can result in the well-known phenomenon of “burnout” [6]. Burnout has been classified as a “state of vital exhaustion” [7]. Burnout is a prolonged response to long-term emotional and interpersonal stressors on the job [8].

Evidence is emerging that the public value accessing local neighbourhoods with green spaces to undertake PA [9], and the public are willing to pay to improve local environments to gain the health benefits of undertaking recreational activities in green spaces [10]. However, a decade of austerity, compounded by the experience of the COVID-19 pandemic, has highlighted the fact that UK outdoor spaces do not have additional appropriate facilities for outdoor safety (such as toilets that are cleaned on a regular basis, handwashing facilities and bins for personal protective equipment (PPE) disposal [11]. The UK Government has put in place unprecedented support through furlough schemes, loans and grants to minimise the likelihood and impact of widespread job losses and resulting scarring of the UK economy [12], but they have not increased funding to improve parks and facilities.

Evidence suggests that obesity-related co-morbidities, such as diabetes and cardiovascular disease, are additional risk factors in the complications of COVID-19 [13]. However, recent UK government advice on reducing obesity to beat COVID-19 has been criticised for not taking into account the impact of PA in green spaces that could improve physical health and well-being [14].

The COVID-19 quarantine requirements, along with social distancing and self-isolation, have perpetuated an environment that promotes physical inactivity. Evidence suggests that physical inactivity is habitually linked with poor outcomes for physical and mental health, leading to chronic conditions and increased risk of mortality [15]. The legacy of the COVID-19 pandemic will extend the current economic inequalities in the UK, with loss of income and unemployment increasing [16].

The aim of this selective scoping review was to investigate the influence of gross income on the public’s engagement in PA beneficial to health, in outdoor green environments. No hypothesis was formed, as the purpose of this review was to understand if income had an influence on people accessing green spaces within local environments to undertake PA. It was conjectured on the basis of a previous review [17] that income may influence PA in green spaces, leading to health inequalities and disparities. To comprehend if there was a correlation or association, a closer examination was considered to enable further understanding and inform health-promoting living for functioning across the life-course. Taking account of income, evidence suggests that there is a requirement within society that all citizens should have a reasonable chance to be healthy, given that health is vital for attainment of well-being, longevity and social and economic opportunities [18]. Taking account of COVID-19, this paper has been augmented with considerations of the impact of the recent pandemic, as there has been limited evidence on the effect of income on engaging in PA in green spaces, linked with multiple lockdowns and restrictions which facilitate physical inactivity.

## 2. Materials and Methods

A selective scoping review was conducted of international literature by three researchers from the Centre for Health Economics and Medicines Evaluation, School of Health Sciences, Bangor University, Bangor, UK. Peer-reviewed articles were sought using health economic databases, including the Cochrane Database and Library, PsycINFO, PubMed, Web of Science, ASSIA, CINAHL, DARE and EED. These databases were searched for articles published between 1 January 1988 and 21 September 2020 [19]. This search was conducted originally for the purpose of examining the economic literature modelling the value of green and blue space. In this process, peer review articles were found which contained an income component and with the assumption that socioeconomic status was a proxy for income, and therefore, the premise for this review [1], was to examine the economic effect of income on accessing green and blue spaces that had ameliorating effects on health and well-being. Therefore, to capture income effects as a deputation of socioeconomic status, the search was amended and conducted in 2020 to capture the syndemic effect of COVID-19. A syndemic is an aggregation of two or more health conditions, which exacerbates the negative effects of the biosocial interface and examines the relationships between many health outcomes [20]. Reference lists, experts and grey literature were also reviewed. Article screening of titles, abstracts and full texts was conducted by three independent reviewers (LHS, ML and RTE) to minimise bias and ensure rigour. Papers meeting the criteria were critically appraised for methodological quality by two independent researchers with a Critical Appraisal Skills Programme checklist [21]. Descriptive thematic analysis was conducted and synthesised to examine the impact of income or socioeconomic status on valuing green spaces for engaging in PA.

## 3. Results

**Figure 1:**
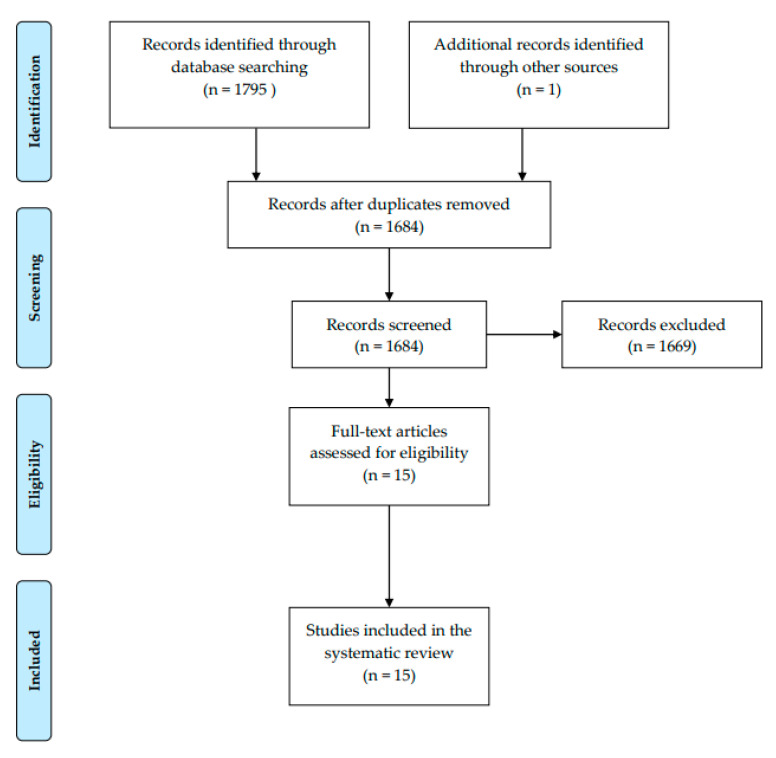
Preferred Reporting Items for Systemic Reviews and Meta-Analyses (PRISMA), 2009 (Moher et al., 2009) flow diagram for systematic review of income and physical activity in green spaces.

A Preferred Reporting Items for Systemic Reviews and Meta-Analyses (PRISMA), 2009 (Moher et al., 2009) flow diagram of the selective scoping review is presented in Figure 1. The search strategy returned 1684 articles, which were screened. After duplicates were removed, and then applying the inclusion and exclusion criteria, 15 articles were included for critical appraisal in this scoping review (see Supplementary File). A similar review was conducted by Lynch et al., 2018 [17]. Characteristics of the included studies are presented in Appendix A, and quality appraisal outcomes for these included papers are presented in Appendix B.

Two themes were identified during the process of data extraction. Ten of the included studies were on the theme of environment and well-being [22,23,24,25,26,27,28,29,30,31], and the remaining five included studies were focused on PA and income/socioeconomic status [32,33,34,35,36]. The studies categorised as “environment and well-being” will be discussed first, followed by the studies on PA and income/socioeconomic status.

### 3.1. Theme 1: Environment and Well-Being

In the first theme, environment and well-being, ten papers were included [22,23,24,25,26,27,28,29,30,31]. A study [22] based in the Washington, DC, area of the United States of America was conducted to explore the relationships between perceptions of neighbourhood environment and sedentary time, as well as between the objective built environment and sedentary time among a population at risk for cardiovascular disease. A total of 176 participants (males and females) took part in the study. Local neighbourhood perception was found to be able to impact on willingness to engage in PA. Negative perceptions of local neighbourhoods such as safety issues, unattractive surroundings and lack of social cohesion can have a negative impact on willingness to engage in PA in local neighbourhoods. Research findings indicated that individuals living in lower-middle-income areas had a negative perception of their local neighbourhoods, which was associated with greater time spent in sedentary activity [22].

A survey based in the city of Seoul, Korea [27] investigated walking and perceptions of social capital levels, in order to study use of public spaces in a walking-friendly urban environment. Results indicated that various urban design techniques should be considered (including trees for shelter) to encourage people to walk for leisure and networking opportunities. In addition, the findings suggested that people who had walked for leisure had higher levels of neighbourly trust and neighbourly networks than those who did not walk for leisure.

A study conducted in South Africa [31] investigated the relationship between exposure to green space and onset of depression. Nationally representative panel data from South Africa were used to investigate the effect of the green living environment on the risk for the occurrence of depression. Sampling of households was based on a stratified two-stage cluster design. In the first stage, 400 of 3000 primary sampling units were included, and in the second stage, two clusters of 12 dwelling units each were drawn from within each primary sampling unit for a total of 24 dwelling units per primary sampling unit. The findings suggested that, in South Africa, individuals who identified as middle-income and resided in a green living environment had a reduced risk of incident depressive symptoms. Within the context of South Africa, with its long history of racially determined income disparity and land tenure inequality, which has persisted during the post-apartheid era, the findings highlighted the importance of green space for its apparent protective effects against onset of depression [31]. These results contribute to a better understanding of how ecosystem services are related to a sense of place and human well-being [31].

### 3.2. Theme 2: Physical Activity and Income/Socioeconomic Status

In the second theme, physical activity and income/socioeconomic status, five papers were included [32,33,34,35,36]. Exploration of the influence of socioeconomic status (SES) on levels of engagement in PA identified inter-related factors associated with SES-influenced levels of PA. Walking for transport is a means of building PA into daily activity; however, transport-related walking is in decline [37]. Evidence has suggested that SES is highly correlated with levels of participation in PA, and SES indicators for PA are less distinct. Research conducted among older people in Hong Kong [24] examined SES indicators of education and individual and area-level household income, perceived neighbourhood characteristics and sense of community, along with benefits of PA, to assess influence on transport-related PA. The results suggested a relationship between SES and walking for transport. Sense of community and perceptions of the quality of local community characteristics, along with higher levels of individual and area-level household income, were also shown to be strongly associated with frequency and level of walking for transport and health-enhancing gains.

These findings are consistent with a study conducted in Canada [29], which examined SES, sense of community and neighbourhood characteristics that could influence people regarding participating in PA. Results indicated that household and neighbourhood-level income, along with education and sense of community, were strongly associated with level of engagement in PA [29]. Higher educational attainment, household income and affluence in community neighbourhoods were significantly associated with greater levels of PA, with increased incidence of inactivity, higher levels of overweight and obesity associated with lower educational and income levels and higher levels of deprivation. It was noted in the findings that there were some gender and age differences associated with SES and levels of PA, with males engaging in more PA than females and older adults less likely to be physically active, depending on perceived local environments. This is a similar conclusion identified in a study undertaken in San Diego, USA [26], which identified that older adults were more likely to engage in PA if their local neighbourhood was safe and pleasant, with greater density of recreational facilities (greenery, parks). Easy access to the coast was also positively associated with greater levels of PA [26]. In addition, higher educational attainment, household income and better general health significantly impacted higher levels of PA among older adults. Higher health status and perceived local environments were shown to significantly increase PA among older adults. The relationship between actual and objective characteristics of local neighbourhoods identified that among older females, local environments that are safe, with greater density of recreational opportunities that contain greener, aesthetically pleasant surroundings, promote greater engagement in PA [30]. However, although SES (income, education) was among the demographic characteristics examined, it was not of significance as the sample was older adults who were not in employment. When examining influences on levels of PA, SES can impact on meeting recommended PA guidelines.

A study conducted in the USA [25] examined social and physical environments that stimulate levels and intensity of PA. Quality and perceptions of local neighbourhoods directly influenced levels of PA and indirectly affected motivation and self-efficacy for engagement in PA [25]. Evidence suggests income level affects motivation levels and intensity of PA, as accessibility to better-quality neighbourhoods which are aesthetically pleasing (including some green spaces) and safe and which have some facilities stimulates both engagement and participation in PA. Housing selection is based on income and associated with neighbourhood quality and access to facilities for PA.

Evidence suggests that there is a disproportionate allocation of relational opportunities between deprived and affluent neighbourhoods [23]. In addition, an examination of residential self-selection noted that greater availability of pay for recreational facilities in local neighbourhoods affected levels and engagement in PA.

SES and level of general health status are inextricably linked and impact on levels of PA. Environments that promote PA can have ameliorating effects on physical, mental and psychological well-being [28]. A study conducted in Austria [28] examined sociodemographic characteristics and levels of PA in outdoor environments, with results indicating that being male, older with a higher income and having a higher level of general health status signified greater levels of participation in PA and lower levels of psychological distress [28].

### 3.3. The Relationship between Income and Exercise in Green and Blue Spaces during the COVID-19 Pandemic Lockdown

The COVID-19 pandemic changed how and where people can be physically active due to government-imposed restrictions on local distancing and ability to access green and blue spaces. The National Trust is the largest conservation charity in Europe, with the aim of protecting coastlines, historic sites, countryside areas and green spaces across the UK, with a commitment to diversity and inclusion. At the start of the COVID-19 pandemic in March 2020, the National Trust closed public access to its sites, but on 17 March 2020, it made the decision to open many of its gardens and parks to the public for free, in recognition that people need access to open space. The importance of outdoor space for mental health was highlighted by the British Broadcasting Corporation (BBC), London, UK. During the next few days, visitors travelled to the coast and countryside to visit National Trust sites [38]. However, on 21 March 2020, following the Government’s announcement of the closure of pubs, restaurants, cafes, gyms and leisure centres, and further travel restrictions, and public health advice, the National Trust made the decision to close all of its gates and parks, as high demand meant that social distancing could not be enforced, putting people at risk of spreading the virus [38]. Similarly, access to National Parks across the UK was restricted to deter people from travelling to those areas and reduce the strain on the National Health Service (NHS). Restricting access to outdoor public spaces is a health equity issue because, unlike higher-income families living in detached housing, people living in apartments, particularly in urban areas, are less likely to have access to green space. Furthermore, there are often fewer green spaces in lower-income areas compared to higher-income areas, and these are often smaller and under-maintained [39,40,41,42], restricting access for people in these areas due to current social distancing rules. In containment of the spread of COVID-19, globally, governments implemented restrictions on citizens regarding self-isolation, travel, and health protection, and made public health information and guidance available to citizens.

A national survey of the impact of the COVID-19 pandemic on movement and play behaviours of Canadian children [42] found that income was associated with more time walking, cycling and playing outside. Children living in a house compared with those living in an apartment may have easier access to green and blue spaces on their doorsteps for outdoor play and PA [42].

As discussed earlier in this paper, the benefits of exercise on mental and physical health are well-documented. The benefit of physical exercise on improving immune functioning is particularly salient during the current COVID-19 pandemic. While social distancing measures are currently in place, and to ensure the safety of the public during the pandemic, extra space is needed for people to safely engage in their PA of choice (e.g., walking, running, cycling, swimming).

## 4. Discussion

The aim of this selective scoping review was to examine how income affects accessing local green space to engage in outdoor PA to improve well-being. We examined the international literature included in this scoping review, then applied a COVID-19 pandemic lens to explore the relationship in depth. The findings from this scoping review demonstrate there is a relationship between income and use of green space, but the relationship is unpredictable and there is still a lack of knowledge about what kind of green spaces are best for health benefits. Urban green areas may provide safer opportunities for outdoor PA, such as neighbourhood walking or cycling. Evidence has indicated that perceptions of local neighbourhoods can have an impact on participating in PA. Negative perceptions of local environments are associated with lower income and sedentary behaviour [22]. However, public green spaces in a walking-friendly urban environment have been shown to facilitate walking for leisure, expansion of social networks and development of social capital [27]. In addition, evidence indicates that interaction with the natural environment in green spaces can have a positive influence on population physical and mental health and well-being. Local green space to engage in outdoor PA to improve well-being is valued by the public [10].

The evidence presented in this scoping review would suggest that SES characteristics are intimately linked with levels of PA and are influenced by neighbourhood characteristics such as walkability [24], sense of community [29], environmental quality [26], density of recreational facilities [30], motivation levels [25] and residential self-selection [23]. All of these can positively or negatively influence levels of PA and general health and well-being status. However, as outlined by this scoping review, there is still limited evidence available on the impact of income on accessing and using green spaces in local communities to improve well-being. The COVID-19 pandemic has been a health and economic shock to society. This interlinked shockwave will have longer-term impacts at an individual, community and societal level with resulting implications for physical and mental health and well-being outcomes in both the short- and long-term due to national and local lockdowns and restrictions, such as the closure of gyms and sport centres. Therefore, a wider systems perspective should be taken when developing strategy and policies to address the economics of health as well as the economics of well-being, and a holistic value-based approach should be adopted to address current crisis management with longer-term effects as to public and systems management. Future research taking a COVID-19 perspective should explore what kind of green spaces are best for health and well-being benefits among the public, particularly in light of restrictions placed by lockdowns on accessing infrastructural PA environments such as sports centres and gyms.

Limitations of this review are that the peer-reviewed studies included were mainly urban and Western studies, and therefore, it was not possible to investigate cultural differences especially in relation to remote/rural/urban lifestyles. In addition, a further limitation is that this was a scoping review using principles of a systematic review to ensure a rigorous approach to examine the evidence available on income as a factor in using green spaces. Scoping studies are an emerging method to synthesise research evidence. Scoping studies are a starting point for a potential systematic review, which would either confirm the relevance or identify potential research questions, or not [43]. The authors conducted this scoping review instead of a systematic review as the purpose of this review was to identify knowledge gaps and clarify concepts to investigate the topic further.

## 5. Conclusions

The physical and mental health benefits of taking part in PA in local neighbourhood environments are a growing area of interest for governments and policy makers internationally who are interested in the well-being of their populations and the wealth of their countries. Minimising the detrimental effect of the COVID-19 pandemic is high on the World Health Organization’s agenda, with social distancing needing to be maintained whilst utilising blue and green spaces sensibly to aid physical health and mental well-being. The findings from this scoping review strongly indicate there is an effect of income on using green spaces for PA, but the relationship is erratic and there are many characteristics of the environment, perceived or otherwise, that influence whether an individual engages in PA in local neighbourhood environments or not. This non-sequential relationship between neighbourhood characteristics and perceptions, along with examining the extent that income affects accessing local green space to engage in outdoor PA to improve well-being, requires further investigation. Given the effect of COVID-19 restrictions at local and national levels, the resulting impact on physical and mental health and well-being status has consequences for post-COVID-19.

## Supplementary Materials

The following are available online at https://www.mdpi.com/1660-4601/17/24/9313/s1, Table S1: Income paper.

## Appendix A

**Table A1:** Characteristics of the studies and the main findings.

	Author and Date	Country of Data Collection	Methodology	Percentage of Females and Males	Age Range	Type of Physical Activity	Main Finding Related to Income/Socioeconomic Status
1.	Ahuja et al., 2018	USA	Community based participatory research (CBPR)	56% Females 44% Males	57–60 years old	Sedentary time rather than physical activity per se.	Low socioeconomic status was correlated with more sedentary behaviour.
2.	Boone-Heinonen et al., 2010	USA	Longitudinal study (waves I and III of National data).	51% Females 49% Males	14.9–15.7 years in Wave I/and 21.3–21.9 years in Wave III	Pay facility availability and public facility availability.	Men in built environments do more moderate vigorous physical activity because of the availability of pay facilities.
3.	Cerin et al., 2009	Australia	A stratified two-stage sampling design	60% Females 40% Males	20–65 years	Walking for transport	People with low socioeconomic status tend to walk less for transport reasons.
4.	Cleland et al., 2010	Australia	Survey	100% Female 0% Male	18–45 years	Leisure Time Physical Activity (LTPA)	Older women in deprived areas tend to do less Leisure Time Physical Activity.
5.	Cohen-Cline et al., 2015	USA	Twin study	68% Females 32% Males	Not stated	General physical activity	This study supports the hypothesis that greater access to residential green space is associated with less depression.
6.	Haughton et al., 2006	USA	Survey	67% Females 33% Males	18–65 years	Current physical activity level	Income may influence physical activity but there is no clear link. Physical environmental factors were associated with walking, moderate-intensity activity, and vigorous-intensity activity; however, the relationships varied by form of activity.
7.	Kerr et al., 2014	USA	Survey	100% Females 0% Males	50–79 years	Walking	Age friendly elements are needed to get more elderly people walking. The study indicated that more intense activity is likely to occur in recreational settings that may be free from traffic (or other hazards).
8.	Kim and Yang, 2017	Korea	Survey	49% Females 51% Males	20–70 years	Neighbourhood walking	Surrounding areas need to be a certain way to encourage people to walk in their neighbourhood. It is necessary to contemplate various urban design techniques (including trees for shelter) that can encourage people to walk for leisure.
9.	Lee, 2007	USA	Telephone survey	54% Females 46% Males	Adults	Physical activities including recreational physical activities and transportation physical activities.	This study found that having high health risk was associate with less physical activity for both recreation and transportation purposes, while being economically challenged was associated with more physical activity for transportation purposes.
10.	Maas et al., 2008	The Netherlands	Interviews	54% Females 46% Males	12 to over 65 years	Physical activity sports and walking for commuting purposes.	Those who live in more green environments do not necessarily walk more for commuting purposes. People with more green space in their living environment do not walk more often for commuting purposes and do not walk for commuting purposes for a longer period.
11.	Mytton et al, 2012	England, UK	Cross-sectional observational study.	56% Females 44% Males	16 years and above	Physical activity levels.	The findings would appear to accord with the national study of all-cause and cardiovascular mortality. This study found that those living in the greenest quintile of England (using the generalised land use database) had lower adjusted mortality. They suggested that this was most likely due to greater physical activity. However, the study could not explain the higher odds of being obese or overweight among those living in the greenest quintile of England found in a similar study.
12.	Niedermeier et al., 2017	Austria	Cross-sectional study	47% Females 53% Males	18 years and above	Mountain physical activity	The prevalence of mental health issues is lower in Austrians engaging in mountain physical activity
13.	Prince et al., 2011	Canada	Cohort study	54% Females 46% Males	18–65 years	Indoor recreation facilities, outdoor–winter, outdoor–summer, park area, bike/walking path, green space (k2) per 1000 people.	Socioeconomic status may have more of an effect on female physical activity level than male physical activity level. The results of this study suggest that in Ottawa, Canada variation in PA and overweight/obesity levels can be attributed to the neighbourhood of residence. Findings suggest that neighbourhood-level interventions that support physical activity and healthy weight control may need to be gender tailored. Furthermore, the recreation environment may play less of a role in physical activity levels, specifically higher intensity physical activity, than access to amenities in the food environment, a possible indicator of mixed land use. The social environment, specifically neighbourhood-level sense of belonging, voting participation and SES may play more important roles in male outcomes, while individual-level SES may be more important for females.
14.	Shin et al., 2011	USA	Cross-sectional study	100% Females 0% Males	55–84 years	Physical activities for older adults and duration	Although the total amount of greenery and the amount of street greenery were found to be important factors that increase older African American women’s physical activity, it is difficult to identify which type of greenery (e.g., trees, shrubs, or grass) has an essential impact on their physical activity.
15.	Tomita et al., 2017	South Africa	Survey	100% Females 0% Males	20 to over 35 years	Exposure to green space	The findings suggest that, in South Africa, African individuals belonging to the middle-income group and residing in a green living environment had a reduced risk of incident depressive symptoms. Within the context of South Africa, with its long history of racially determined income disparity and land tenure inequality, the findings highlight the importance of green space for its apparent protective effects against onset of depression.

## Appendix B

**Table A2:** Quality appraisal outcomes.

	Author and Date	Statistical Tests Conducted	Proportions of Missing/Incorrect Data Reported	Response Rates Reported	Inclusion/Exclusion Criteria Explicitly Stated
1.	Ahuja et al., 2018	Student’s t test and Factor Analysis	No missing data reported	No response rate reported	No
2.	Boone-Heinonen et al., 2010	Regression analysis	Yes	No response rate reported	Yes
3.	Cerin et al., 2009	Product coefficient test performed using bootstrapping re-sampling techniques	No missing data reported	12% response rate	No
4.	Cleland et al., 2010	Chi-square, ANOVA and Regression analyses	Yes	54% response rate	No
5.	Cohen-Cline et al., 2015	Multilevel random intercept model	No	No response rate reported	Yes
6.	Haughton et al., 2006	Structural equation modelling (SEM) using LISREL for Windows, version 8.52	Yes	No response rate reported	Yes
7.	Kerr et al., 2014	Linear regression models	No	No response rate reported	No
8.	Kim and Yang, 2017	One-way ANOVA and regression models	No	24% response rate	No
9.	Lee, 2007	ANOVA and Pearson correlation analysis	No	34% response rate	Yes
10.	Maas et al., 2008	Multilevel logistic regression analysis	No	No response rate explicitly stated, but 4899 answered all vital questions.	No
11.	Mytton et al, 2012	Logistic regression was used to test for an association between physical activity and green space (in quintiles)	No	No response rate reported	Yes
12.	Niedermeier et al., 2017	Multiple linear regression analyses	Yes	No response rate reported	No
13.	Prince et al., 2011	Chi-square, binary logistic regression models, a six–step modelling strategy	Yes	The response rates for the survey years are as follows: 60% (2003); 59% (2004); 64% (2005); 66% (2006); and 59% (2007).	No
14.	Shin et al., 2011	Correlational analyses and Chi-square	Yes	34% response rate	Yes
15.	Tomita et al., 2017	Logistic regression models	No	No response rate reported	Yes
