# Supplementary material for: A Scoping Review of How Income Affects Accessing Local Green Space to Engage in Outdoor Physical Activity to Improve Well-Being: Implications for Post-COVID-19
Source: Int J Environ Res Public Health. 2020 Dec 12;17(24):9313. doi: 10.3390/ijerph17249313 (PMC7764517; doi:10.3390/ijerph17249313)
Supplement: Supplementary file 1 [file ijerph-17-09313-s001.pdf]

Supplementary File - 15 included income papers:

	Author and date	Study title	Theme
1.	Ahuja et al., 2018	Examining relationships between perceptions and objective assessments of neighborhood environment and sedentary time: Data from the Washington, D.C. Cardiovascular Health and Needs Assessment	Environment and well-being
2.	Boone-Heinonen et al., 2010	Residential self-selection bias in the estimation of built environment effects on physical activity between adolescence and young adulthood	Environment and well-being
3.	Cerin et al., 2009	Explaining socio-economic status differences in walking for transport: An ecological analysis of individual, social and environmental factors	Environment and well-being
4.	Cleland et al., 2010	Individual, social and environmental correlates of physical activity among women living in socioeconomically disadvantaged neighbourhoods	Physical activity and income/socioeconomic status
5.	Cohen-Cline et al., 2015	Access to green space, physical activity and mental health: a twin study	Physical activity and income/socioeconomic status
6.	Haughton et al., 2006	Individual, social environmental, and physical environmental influences on physical activity among black and white adults: a structural equation analysis.	Environment and well-being
7.	Kerr et al., 2014	Neighborhood Environment and Physical Activity among Older Women: Findings from the San Diego Cohort of the Women's Health Initiative	Environment and well-being
8.	Kim and Yang, 2017	Neighborhood walking and social capital: The correlation between walking experience and individual perception of social capital	Environment and well-being
9.	Lee, 2007	Environment and active living: The roles of health risk and economic factors.	Physical activity and income/socioeconomic status
10.	Maas et al., 2008	Physical activity as a possible mechanism behind the relationship between green space and health: A multilevel analysis	Physical activity and income/socioeconomic status

	Author and date	Study title	Theme
11.	Mytton et al, 2012	Green space and physical activity: An observational study using Health Survey for England data	Physical activity and income/socioeconomic status
12.	Niedermeier et al., 2017	Prevalence of mental health problems and factors associated with psychological distress in mountain exercisers: A cross-sectional study in Austria	Environment and well-being
13.	Prince et al., 2011	A Multilevel Analysis of Neighbourhood Built and Social Environments and Adult Self-Reported Physical Activity and Body Mass Index in Ottawa, Canada	Environment and well-being
14.	Shin et al., 2011	The distance effects of environmental variables on older African American women's physical activity in Texas	Environment and well-being
15.	Tomita et al., 2017	Green environment and incident depression in South Africa: a geospatial analysis and mental health implications in a resource-limited setting	Environment and well-being
